# Genomic characterization of *Salmonella* Cerro ST367, an emerging *Salmonella* subtype in cattle in the United States

**DOI:** 10.1186/1471-2164-15-427

**Published:** 2014-06-04

**Authors:** Lorraine D Rodriguez-Rivera, Andrea I Moreno Switt, Lovorka Degoricija, Rixun Fang, Craig A Cummings, Manohar R Furtado, Martin Wiedmann, Henk C den Bakker

**Affiliations:** Department of Food Science, Cornell University, Stocking Hall, Ithaca, NY 14853 USA; Life Technologies Corporation, 180 Oyster Point Blvd, South San Francisco, CA 94080 USA; Facultad de Ecología y Recursos Naturales, Universidad Andres Bello, Santiago, Chile; Genentech, 1 DNA Way, South San Francisco, CA 94080 USA; Biology for Global Good LLC, 420 Ventura Pl, San Ramon, CA 94583 USA

## Abstract

**Background:**

Within the last decade, *Salmonella enterica* subsp. *enterica* serovar Cerro (*S.* Cerro) has become one of the most common serovars isolated from cattle and dairy farm environments in the northeastern US. The fact that this serovar is commonly isolated from subclinically infected cattle and is rarely associated with human disease, despite its frequent isolation from cattle, has led to the hypothesis that this emerging serovar may be characterized by reduced virulence. We applied comparative and population genomic approaches to (i) characterize the evolution of this recently emerged serovar and to (ii) gain a better understanding of genomic features that could explain some of the unique epidemiological features associated with this serovar.

**Results:**

In addition to generating a *de novo* draft genome for one *Salmonella* Cerro strain, we also generated whole genome sequence data for 26 additional *S.* Cerro isolates, including 16 from cattle operations in New York (NY) state, 2 from human clinical cases from NY in 2008, and 8 from diverse animal sources (7 from Washington state and 1 from Florida). All isolates sequenced in this study represent sequence type ST367. Population genomic analysis showed that isolates from the NY cattle operations form a well-supported clade within *S.* Cerro ST367 (designated here “NY bovine clade”), distinct from isolates from Washington state, Florida and the human clinical cases. A molecular clock analysis indicates that the most recent common ancestor of the NY bovine clade dates back to 1998, supporting the recent emergence of this clone.

Comparative genomic analyses revealed several relevant genomic features of *S.* Cerro ST367, that may be responsible for reduced virulence of *S.* Cerro, including an insertion creating a premature stop codon in *sopA*. In addition, patterns of gene deletion in *S.* Cerro ST367 further support adaptation of this clone to a unique ecological or host related niche.

**Conclusions:**

Our results indicate that the increase in prevalence of *S*. Cerro ST367 is caused by a highly clonal subpopulation and that *S.* Cerro ST367 is characterized by unique genomic deletions that may indicate adaptation to specific ecological niches and possibly reduced virulence in some hosts.

**Electronic supplementary material:**

The online version of this article (doi: 10.1186/1471-2164-15-427) contains supplementary material, which is available to authorized users.

## Background

Genomic characteristics associated with the emergence or reemergence of pathogens in livestock operations can be subdivided into two categories; (i) genomic features that increase the adaptation to a host, or facilitate the jump to a new host species, or (ii) genomic features that provide increased adaptation to environmental factors in the livestock environment, such as antibiotic resistance. Comparative and population genomic studies are particularly suited to determine which features are responsible for the emergence of certain pathogens. For instance, Price et al. [1] showed that a putative host jump, from humans to livestock, in a clonal complex in *Staphylococcus aureus* was associated with the loss of phage-carried human virulence genes and with the acquisition of tetracycline and methicillin resistance.

*Salmonella enterica* is one of the most frequent causes of bacterial foodborne illness and death in the United States [2]. In *Salmonella*, examples of emergent clones include *S.* Typhimurium DT 104, a multidrug resistant clone, which has seen a global epidemic spread from 1990 [3], and *S. enterica* serovar 4,5,12:i:–, a monophasic variant of *S.* Typhimurium, which showed a global increase in the mid-1990s [4]. In this study, we present comparative and population genomic research on *S. enterica* subsp. *enterica* serovar Cerro (*S.* Cerro). *S.* Cerro is rarely associated with human disease, with only one outbreak reported in the US so far that could be solely attributed to this serovar [5]; an additional outbreak was recently reported and it was linked to multiple serovars, including *S.* Cerro [6]. However, this *Salmonella* serovar has emerged over the last decade as one of the most abundant *Salmonella* serovars in cattle operations in the northeastern US [7], including one of the most common serovars among subclinical dairy cattle and in the dairy farm environment [8] in the northeastern United States. Most of the *S*. Cerro isolated from cattle and farms represent one pulsed field electrophoresis (PFGE) type, indicating that a single clonal lineage is involved in this emergence [7]. It is unknown what causes *S.* Cerro to be associated with cattle and why it is rarely involved in human disease. Therefore, we hypothesize that *S.* Cerro has distinct genomic characteristics that explain its association with cattle and limited association with human disease.

## Results and discussion

### De novo assembly shows that *S.* Cerro FSL R8-0235 has a genome size of approximately 4.7 Mbp, contains six prophage regions and represents MLST sequence type ST367

After exclusion of contigs fewer than 200 bp, the total length of the *S.* Cerro FSL R8-0235 draft *de novo* assembly was 4,675,817 bp. The assembly consisted of 126 contigs, with a contig N50 of 292,947 bp, and a maximum contig length of 691,181 bp. The average coverage depth of the assembly was 96X. One contig, contig 016, contained genes of an IncI1-like plasmid, however it is unclear whether this is an integrated or extrachromosomal plasmid. In addition to genes involved in plasmid transfer, stability and replication, this plasmid also carries genes encoding a resistance nodulation division (RND) efflux pump [9]. However, none of the isolates sequenced in this study showed resistance to single or multiple antimicrobial agents. No evidence for the existence of additional plasmids within the genome was found. This may be at least partially due to the presence of a DNA phosphorothioation-dependent restriction modification (RM) system in all *S.* Cerro strains examined in this study. While this RM system has been well characterized in *S.* Cerro [10], a PSI-BLAST search reveals this type of RM system is very rare among *Salmonella*, and only found in a limited number of sequenced *Salmonella* strains of serovars Saintpaul (SARA23, str. 9712, str. JO2008), Namur (str.05-2929) and Panama (ATCC 7378).

Prediction of lysogenic prophages and prophage remnants in the *S.* Cerro FSL R8-0235 genome was performed using Prophinder [11]. Six putative prophages or remnants of prophages, ranging in length from 5.78 to 31.52 Kb, were predicted to be present in the *S.* Cerro FSL R8-0235 genome (Table 1). The six prophage regions, which we refer to as prophage 57014, 57017, 57018, 57023, 57024, and 57025, were compared, using RAST [12], to previously sequenced genomes to identify homologous regions. Prophages 57023 and 57025 (Table 1) are similar in composition to a *S.* Typhimurium ES18-like bacteriophage, while 57014 shows similarity to an Enterobacteria P22-like prophage. While typical *Salmonella*-associated prophages, such as Gifsy-1, Gifsy-2, Fels-1, and Fels-2 [13–15] were not predicted to be present in the *S.* Cerro FSL R8-0235 genome, prophage 57024 shared many genes with a prophage found in *Photorhabdus luminescens* subsp. *laumondii* TT01, which has been described as a successful insect pathogen as well as symbiont of soil entomopathogenic nematodes [16].

**Table 1:** **Prophage distribution in the** ***Salmonella*** **Cerro FSL R8-0235 genome**

Prophage ID	Contig^a^	Length	Previously described phages similar to ***S.*** Cerro prophages
57018	003	5,780 bp	Putative prophage remnant, found in *E. coli*, *S.* Typhi (CT18), *S.* Newport (SL254)
57024	009	27,456 bp	Similar to prophage in *S.* Baildon (FSL R6-199) as well as prophages in *E. coli* and *Photorhabdus luminescens* subsp. *laumondii* TT01
57025	009	31,520 bp	*S.* Typhimurium bacteriophage ES18-like, similar prophages in *S.* Senftenberg (FSL A4-543), *S.* Schwarzengrund (CVM19633), and *S.* Montevideo (FSL S5-403)
57023	013	15,396 bp	*S*. Typhimurium bacteriophage ES18-like, similar prophages in *S.* Senftenberg (FSL A4-543) and *S.* Choleraesuis (SC-B67)
57017	017	7,296 bp	Putative prophage remnant, found in a wide variety of *Salmonella enterica* serovars, *E. coli* and *Shigella*
57014	018	11,952 bp	Enterobacteria P22 phage, similar prophages are found in *S.* Dublin (CT_02021853), *S.* Paratyphi A (ATCC 9150, AKU_12601)

^a^Contig in the *S.* Cerro pseudogenome where the predicted prophage is encoded.

Genome assembly based multi locus sequence typing (MLST) was performed using the online tool [17] of the Center for Genomic Epidemiology (Lyngby, Denmark; http://www.genomicepidemiology.org/) and an additional BLASTN search. This analysis revealed that *S*. Cerro FSL R8-0235 belongs to sequence type (ST) 367. According to the *Salmonella* MLST database (http://mlst.warwick.ac.uk/mlst/dbs/Senterica) ST367 is associated with a *S.* Cerro isolate from a human case in Germany in 1985. The database also contains an accession of the type strain of *S.* Cerro, isolated from swine in 1936 in Uruguay. This strain belongs to ST1291 and displays a different allelic type at each of the seven MLST loci. *S.* Cerro therefore is very likely to be polyphyletic, which makes interpretation of historical references without genomic or MLST sequence data difficult. Because all isolates sequenced in this study belong to ST367, we will refer to these isolates as *S.* Cerro ST367 from here on. Timme et al. [18] recently published sequence data for another *S.* Cerro ST367 strain (strain 818; NZ_AOZJ00000000); this group showed that, among all serovars that have been sequenced so far, *S.* Adelaide FSL A4-669 is most closely related to *S.* Cerro ST367 which is consistent with our study (see below).

### Population genomic analysis of 27 *Salmonella* serovar Cerro isolates suggests a recent clonal expansion of a bovine-associated *S.* Cerro lineage

**Figure 1:**
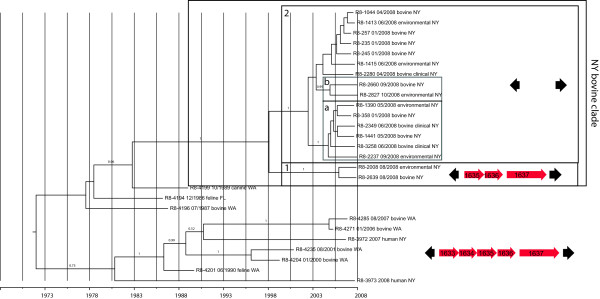
**Tip-dated phylogeny of the 27** ***S***. **Cerro isolates sequenced in this study with stepwise deletion of a D-alanine transporter encoding gene cluster mapped onto the phylogeny.** Values on the branches represent posterior probabilities. Genes adjacent to the D-alanine transporter encoding gene cluster are represented as black arrows, genes in the cluster are represented as red arrows. Numbers in the arrows refer to STM gene tags as used in the genome sequence of *S.* Typhimurium LT2. Labels on the tips indicate isolate accession numbers, isolate date (month/year) and source.

To infer whether the *S.* Cerro isolates associated with bovine hosts and cattle-associated environments form separate subpopulations from *S.* Cerro isolated from other sources, we obtained whole genome sequencing data for 26 additional isolates (Table 2). After removal of putative recombinogenic regions, as identified by BratNextGen [19], and SNPs that were present in fewer than 90% of the isolates, 343 SNPs were left for analysis. To assess the presence of a temporal signal in the dataset, a Path-O-Gen (available from http://tree.bio.ed.ac.uk/software/pathogen/) analysis was performed using a maximum likelihood tree inferred from the SNP data set. This analysis showed a correlation (Pearson’s Correlation Coefficient 0.80, *R*^2^ = 0.645) between the time of isolation of the individual isolates and the root-to-tip divergence, indicating a temporal signal for this dataset and justifying a molecular clock based phylogenetic analysis. A Bayesian analysis, assuming a relaxed molecular clock and a constant population size, inferred the mean mutation rate for the core genome of the 27 *S*. Cerro isolates to be 2.4 × 10^−7^/site/year (95% Highest Probability Density (HPD) 1.5 × 10^−7^ – 3.3 × 10^−7^). This mutation rate is comparable to mutation rates estimated for *Buchnera aphidicola*[20] and *Helicobacter pylori*[21], but about twice as fast as recently inferred for *S.* Agona [22]. The New York bovine isolates are found in a well-supported (posterior probability 1.0) clade (NY bovine clade; see Figure 1), well separated from the isolates from Washington state, Florida, and the human clinical isolates from New York state. This may indicate that, although isolates of *S.* Cerro of the bovine-associated clade were prevalent in farm environments, and thus farm personnel would be frequently exposed to this clone, this clone was not responsible for the human cases in New York state represented by these two isolates. The time of the emergence of the most recent ancestor (MRCA) of the NY bovine clade is estimated to be 1998 (95% HPD 1991–2003). The NY bovine clade is further split up into two clades: (i) a clade with two isolates from northeastern New York (Figure 1: clade 1) and (ii) a clade with 15 bovine associated isolates from western NY state (Figure 1: clade 2). The MRCA of the latter clade dates back to 2002 (95% HPD 1999–2005). Within clade 2, two well supported clusters were identified (marked ‘a’ and ‘b’ in Figure 1). Specifically, ‘cluster a’ contains six isolates that were isolated from Steuben county (NY) and the neighboring Livingston county (NY). This finding suggests a phylogeographic signal in the dataset, which should facilitate more detailed tracing of the emergence of *S*. Cerro ST367 throughout the northeastern US with a larger sampling and a population genomic analysis.

**Table 2:** **27** ***Salmonella*** **Cerro isolates sequenced in this study**

FSL no.^a^	Source	Date of isolation	Obtained from^b^	County and/or state of origin	SRA accession^c^
R8-4199	Canine host	Oct-1989	WSU	WA	SRR654177
R8-4201	Feline host	Jun-1990	WSU	WA	SRR654178
R8-4194	Feline host	Dec-1986	WSU	FL	SRR654174
R8-4196	Bovine host	Jul-1987	WSU	Grant, WA	SRR654176
R8-4235	Bovine host	Aug-2001	WSU	Yakima, WA	SRR654180
R8-4285	Bovine host	Aug-2007	WSU	Yakima, WA	SRR654183
R8-4271	Bovine host	Jan-2006	WSU	Grant, WA	SRR654182
R8-4204	Bovine host	Jan-2000	WSU	Yakima, WA	SRR654179
R8-3973	Human host	2008	NYSDOH	NY	SRR653053
R8-3972*	Human host	2007	NYSDOH	NY	SRR653052
R8-2827*	Farm environment	Oct-2008	CU-Warnick	Tompkins, NY	SRR653002
R8-2660*	Bovine host, non-clinical	Sep-2008	CU-Warnick	Niagara, NY	SRR654036
R8-2280*	Bovine host, clinical	Apr-2008	CAHDC	Wyoming, NY	SRR653929
R8-0257	Bovine host, non-clinical	Jan-2008	CU-Warnick	Genesee, NY	SRR653610
R8-1413	Farm environment	Jun-2008	CU-Warnick	Niagara, NY	SRR653005
R8-1441	Bovine host, non-nlinical	May-2008	CU-Warnick	Steuben, NY	SRR653928
R8-0358	Bovine host, non-clinical	Jan-2008	CU-Warnick	Steuben, NY	SRR653721
R8-0245	Bovine host, non-clinical	Jan-2008	CU-Warnick	Genesee, NY	SRR653609
R8-2349	Bovine host, clinical	Jun-2008	CAHDC	Livingston, NY	SRR653931
R8-1415	Farm environment	Jun-2008	CU-Warnick	Niagara, NY	SRR653010
R8-2008	Farm environment	Aug-2008	CU-Warnick	Franklin, NY	SRR653009
R8-2639	Bovine host, non-clinical	Aug-2008	CU-Warnick	Franklin, NY	SRR654035
R8-3258	Bovine host, clinical	Jul-2008	CAHDC	Livingston, NY	SRR654173
R8-1044	Bovine host, non-clinical	Apr-2008	CU-Warnick	Genesee, NY	SRR653927
R8-2237	Farm environment	Sep-2008	CU-Warnick	Steuben, NY	SRR652998
R8-0235	Bovine host, non-clinical	Jan-2008	CU-Warnick	Wyoming, NY	SRR654552
R8-1390	Farm environment	May-2008	CU-Warnick	Steuben, NY	SRR652996

^a^Isolates marked with an asterisk were used in the Caco-2 invasion assays.

^b^WSU = Washington State University; NYSDOH = New York State Department of Health; CU-Warnick = Cornell University, Warnick laboratory; CAHDC = Animal Health Diagnostic Center, Cornell University.

^c^SRA = Sequence Read Archive (http://www.ncbi.nlm.nih.gov/sra).

### Genome sequence analysis reveals a stepwise evolution, of *S.* Cerro ST367 to a bovine-associated clade, characterized by deletion of selected operons and acquisition of a premature stop codon in *sopA*

Loss or gain of genes within bacterial populations may indicate niche adaption of bacterial subpopulations [23]. To infer patterns of gene loss, we mapped reads of the 27 *S.* Cerro isolates against well-annotated genomes such as those of *S.* Typhimurium LT2, *S.* Typhi CT18, and *S*. Choleraesuis SC-B67. In addition we mapped the reads of *S.* Adelaide FSL A4-669 [24] against these genomes, to determine if the patterns of absence were also observed in the most recent common ancestor of this serovar and the *S.* Cerro population studied here. Reads of the 27 *S.* Cerro isolates mapped to 86, 88, and 90% of the coding sequences in *S.* Typhi CT18, *S.* Typhimurium LT2, and *S.* Choleraesuis SC-B67, respectively. This is very similar to the percentage of genes shared (89%) between *S.* Typhimurium LT2 and *S.* Typhi CT18 [25] and falls in the higher end of the range observed by Jacobsen et al. [26] for a wide variety of *Salmonella* serovars. The genome size, and the high number of shared genes thus suggest that the lineage of *S.* Cerro studied here did not experience notable genome reduction.

Mapping of sequence reads of the isolates of the *S. Cerro* population further revealed a pattern of gene absence generally conserved within the *S.* Cerro population sampled here, suggesting that most of the genomic characteristics associated with the emergence of *S*. Cerro among bovine-associated habitats were present in the MRCA of this *S.* Cerro clade. Interestingly, loss of some SPIs (*Salmonella* Pathogenicity Islands) that were found here to be absent or partially absent (gene deletions) from the Cerro population studied (i.e., ST367), but are present in *S.* Typhimurium LT2 or *S.* Typhi CT18, have been associated with attenuation of virulence. Specifically, the genomic island at *S.* Typhi SPI-10 locus is completely absent from the *S*. Cerro ST367 isolates examined here; this SPI has been associated with virulence in mice [27]. Chaudhuri et al. [28] also showed that significant reduction of fitness of *S.* Typhimurium SL1344 is observed during intestinal colonization of cattle when genes in SPI-10 (in particular STM4489) are disrupted by transposon insertion. Genes homologous to (i) STM2230.1c to STM2240 of SPI-12, and (ii) STM3117, STM3123, and STM3119 to STM3121 of SPI-13 were also found to be absent from *S.* Cerro ST367; these SPIs have been associated with systemic infection of mice in *S*. Typhimurium [29], and replication in macrophages (SPI-13: [30]). Furthermore, disruption of STM2231 in SPI-12 and STM3123 in SPI-13 was previously shown to cause significant reduction in fitness in *S.* Typhimurium SL1344 during intestinal colonization of cattle [28]. In addition, homologs of STM0293, STM0294 and STM0299 are deleted in *S.* Cerro ST367. These genes are found in SPI-16, a SPI associated with intestinal persistence in mice [31]. Disruption of STM0293 in *S.* Typhimurium has been shown to cause reduced fitness with regard to intestinal colonization of cattle [28]. Most of the SPI-related genes found to be absent in *S.* Cerro ST367 were confirmed to be present in *S.* Adelaide FSL A4-669, suggesting loss of these genes/SPIs occurred after the divergence of *S*. Adelaide from the most recent common ancestor of *S.* Cerro ST367. We found evidence for the presence of four complete toxin-antitoxin (TA) modules (STM 2954.1 N-2955.S; STM4030.S-4031; STM3777-78 and STM4449-50) within the *S.* Cerro genomes studied here. This is interesting as De la Cruz et al. [32] suggested that TA modules in *Salmonella* play a role in virulence, and that the number of genomically encoded TA modules is correlated with pathogenicity of individual strains. By comparison, the number of TA modules in *S. enterica* subsp. *enterica* ranges from 5 (*S.* Paratyphi B SPB7) to 10 (*S.* Typhimurium LT2), making *S.* Cerro ST367 one of the subsp. *enterica* serotypes with the lowest number of TA modules. The number of TA modules in *S*. Cerro ST367 is similar to that observed in *Salmonella enterica* subsp. *arizonae*, a subspecies which is predominantly found in cold blooded hosts and does generally not seem to cause illness in warm blooded hosts [33]. Complete or partial absence of some SPIs in all *S.* Cerro ST367 and the low number of TA modules in the genome, thus suggests a putative shift of *S.* Cerro in host and/or tissue tropism before the emergence of the NY bovine-associated clade.

The hypothesis that the *S.* Cerro population studied here shows unique host and/or tissue tropism characteristics is also supported by the finding that all 27 *S*. Cerro ST367 isolates sequenced here were found to carry a premature stop codon in *sopA*, causing a truncation of the gene from 782 aa (in *S*. Typhimurium LT2) to 433 aa. Previous studies have shown that SopA is involved in virulence during bovine gastrointestinal infections by *S.* Typhimurium and *S*. Dublin [34, 35], and that *sopA* mutations are implicated in reduced polymorphonuclear (PMN) cell migration [34, 36], and fluid secretion in ileal loops in calves [34]. Premature stop codons in *sopA* have been found in *S*. Typhi, *S*. Paratyphi A, and *S.* Gallinarum and it has been suggested that loss of a functional SopA has been an important factor in the virulence and adaptation of these serovars to a systemic niche in certain hosts [37, 38].

**Figure 2:**
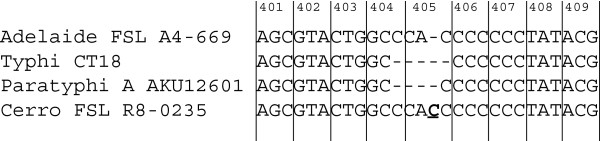
**Alignment of** ***sopA*** **in** ***S***. **Cerro and selected other** ***Salmonella*** **serovars showing premature stop codon in Cerro and** ***sopA*** **polymorphisms in other** ***Salmonella*** **strains and serovars.** Numbers above the alignment indicate the amino acid residues as found in *sopA* in *S.* Typhimurium LT2. *sopA* for *S*. Adelaide FSL A4-669 is in frame, while *sopA* for *S.* Typhi CT18 and *S.* Paratyphi A AKU 12601 show a four and three bp deletion in this region in this region, respectively. *S.* Cerro has a one bp insertion (underlined), leading to a frame shift and premature stop.

Interestingly, the one base-pair insertion responsible for the premature stop codon occurs within a ~10 bp region of *sopA* that also contains deletions in *S*. Typhi and *S*. Paratyphi A (Figure 2). While *S*. Typhi and *S*. Paratyphi A contain additional mutations that may have caused loss of function of SopA [38], the occurrence of deletions in the same region in *S.* Cerro *sopA* suggests this is a replication error prone region in the genome. A conserved domain search (http://www.ncbi.nlm.nih.gov/Structure/cdd/wrpsb.cgi) against the Conserved Domain Database [39] of the aa sequence of the truncated SopA in *S.* Cerro ST367 revealed the premature stop is situated in the SopA central domain [40] of the gene. Furthermore, the truncated SopA protein lacks the capsase-3 cleavage sites, which have been demonstrated to be important in induction of PMN transepithelial migration in *S.* Typhimurium [36]. Although specifically the disruption of the main functional domain in SopA and the loss of the capsase-3 cleavage sites suggest loss of function of SopA in *S*. Cerro ST367, further molecular genetic experiments have to be conducted to reveal if truncation of SopA in *S*. Cerro ST367 has lead to loss of function of this gene, and how it affects host cell invasion (as suggested by Raffatelu et al. [41]) and other SopA associated aspects of *Salmonella* virulence.

Read mapping also showed one gene cluster to be stepwise deleted in the NY bovine clade (Figure 1). This gene cluster contains homologs of the *S.* Typhimurium LT2 genes STM1633 to STM1637. This gene cluster encodes a D-alanine transporter and has been recently shown to be required for intracellular survival in murine macrophage-like cells [42], and disruption of STM1637 has been shown to cause a significant reduction in fitness in intestinal colonization in cattle in *S.* Typhimurium [28]. This gene cluster is present in all 10 Cerro ST367 isolates that do not belong to the bovine clade. Two isolates (FSL R8-2008, FSL R8-2639) lack two genes (STM1633, STM1634) in this gene cluster. These two isolates represent a clade that split off early from the remaining NY bovine-associated-population. The remaining 15 isolates in this clade lack the entire gene cluster (Figure 1). The (partial) absence of the D-alanine transporter gene cluster is currently the only genomic feature that differentiates the NY bovine clade from the remaining population (including isolates from the NY human cases).

### *S.* Cerro displays reduced invasiveness of human epithelial cells compared to other *Salmonella* serovars commonly isolated from bovine sources

**Figure 3:**
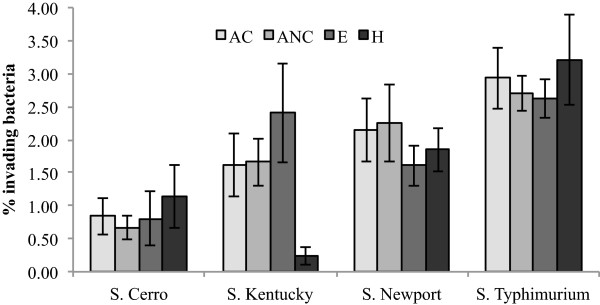
**Caco-2 invasion efficiencies among** ***Salmonella*** **serovars Cerro, Kentucky, Newport, and Typhimurium.** Cells were inoculated at a MOI of 10 and the invasion assays were performed at 37°C and 5% CO_2._ Invasion efficiency was calculated as [CFU recovered/CFU infected] × 100. Data represent the mean of at least three biological replicates, and the error bars represent the standard deviation. The invasion efficiencies for each serovar were analyzed using one-way analysis of variance (ANOVA) and Tukey’s post hoc test, after the data was log-transformed to satisfy ANOVA assumptions of normality. Isolate sources are abbreviated as AC, Animal Clinical; ANC, Animal Non-clinical; E, Environmental; H, Human.

The comparative genomic analyses described above suggest *S.* Cerro lacks several functional genes and genomic elements that are involved in invasion and intracellular survival. To assess if strains of *S.* Cerro ST367 population (Table 2) studied here are impaired in their ability to invade human intestinal epithelial cells, Caco-2 cells were infected with *S.* Typhimurium (*n* = 4), *S.* Newport (*n* = 4), *S.* Kentucky (*n* = 4), and *S.* Cerro (*n* = 4). Each serovar was represented by one isolate each from a bovine clinical case, a subclinically infected bovine host, an environmental sample and a human clinical case. *S.* Cerro isolates were significantly less invasive than isolates of serovars Typhimurium (*P* < 0.0001) and Newport (*P* < 0.0001), but not significantly different from *S.* Kentucky (*P* = 0.0734) (Figure 3). However, the overall invasiveness of *S.* Kentucky seems to be skewed by the presence of one isolate from a human clinical case, which shows very low invasion. When this outlier is excluded from the analysis, the *S.* Cerro isolates are also significantly less invasive than *S.* Kentucky (p = 0.004). Thus, consistent with our genomic analyses, *S.* Cerro ST367 seems to be less invasive in human intestinal epithelial cells than the serovars examined here. Future studies on the ability of *S.* Cerro to invade bovine intestinal epithelial cells and to cause illness in cattle will be necessary though to determine whether *S*. Cerro or specific subtypes within *S*. Cerro truly show attenuated bovine virulence.

## Conclusions

Comparative genomic analyses of 27 *Salmonella* Cerro isolates indicate that this serovar lacks several genes that have previously been shown to be involved in the ability of *Salmonella* serovars to cause intestinal infection. Reduced invasion of human intestinal epithelial cells, as compared to other serovars, further supports the reduced ability of this serovar to cause intestinal infection, however, further experiments are necessary to determine which genes are responsible for this phenotype. Altogether, these results suggest that the emergence of *S.* Cerro ST367 among livestock operations in the northeastern United States may not be due to increased adaptation to the bovine host, nor to increased antibiotic resistance. Instead, the frequent isolation of this serovar on cattle farms [8] may reflect that this serovar was able to disperse rapidly as no efforts were undertaken to control its spread (possibly due to a lack of clinical signs, which left infections undetected). Alternatively, or in addition, *S.* Cerro (or some subtypes within *S.* Cerro) may have unique phenotypic characteristics that were not discovered through the comparative genomic analyses conducted here, but that facilitate environmental survival or dispersal.

## Methods

### Isolates selection

The 27 *S.* Cerro isolates for genome sequencing (*n = 1*) and re-sequencing (*n = 26*) were isolated from 1986 to 2008 from human cases and domesticated and wild animals in 3 different states (i.e., New York, Washington, and Florida; Table 2).

### Genome sequencing, assembly and annotation

The genome of *S.* Cerro FSL R8-0235 was sequenced using the SOLiD™ system (Applied Biosystems, Foster City). Mate-paired 50 bp reads were obtained and a *de novo* assembly was performed as detailed in Den Bakker et al. [24]. Contigs longer than 200 bp were submitted to the NCBI Prokaryotic Genomes Automatic Annotation Pipeline (PGAAP) [43] for automated annotation. Unpaired 50 bp reads for the genomes of the additional 26 *S*. Cerro ST367 isolates were obtained using the SOLiD™ system (Applied Biosystems, Foster City) as detailed in Den Bakker et al. [44].

### Prophage identification

PROPHINDER [11] was used to find putative prophages. The prophage regions were compared, using RAST [12], to previously sequenced genomes to identify homologous regions.

### SOLiD™ read mapping, population genetics analysis, and read mapping based gene presence/absence analysis

SOLiD™ reads were mapped against a reference genome (FSL R8_0235) using PerM [45]. ComB [46] was used to for SNP calling and creation of consensus sequences. Regions with coverage less than 10X were masked in the consensus sequences. Consensus sequences created with ComB were used as input for the BratNextGen [19] recombination detection software, using 100 replicates of 50 iterations each. SNPs in regions that were predicted to be involved in a recombination event with *P* < 0.01 were excluded from the analysis.

A maximum likelihood (ML) tree based on the SNP data was created in MEGA 5 [47], and this ML tree was used to test for the presence of a temporal signal in the dataset using Path-O-Gen 1.4 (available from http://tree.bio.ed.ac.uk/software/pathogen/). BEAST version 1.7.5 [48] was used to create a tip-dated phylogeny of the *S*. Cerro isolates. Four different models differing in assumptions on mutation rate and effective population size (strict clock, constant population size; strict clock, Gaussian Markov random field (GMRF) model [49]; relaxed clock, constant population size; relaxed clock, GMRF model) were run for 10 million generations each and compared using the Bayes factor as implemented in Tracer version 1.5 (A. Rambaut available from http://tree.bio.ed.ac.uk/software/tracer/).

Read mapping based gene presence/absence analysis was performed by mapping SOLiD™ reads to selected reference genomes using PerM [45]. Coverage per annotated gene feature in the reference genome was subsequently obtained using the ‘coverage’ tool from the BEDtools suite [50].

### Caco-2 cell invasion assays of *S.* Cerro, *S.* Kentucky, *S.* Typhimurium, and *S.* Newport

To compare the ability of *S.* Cerro isolates to invade human intestinal epithelial cells, Caco-2 cells were infected with *S.* Typhimurium (*n* = 4), *S.* Newport (*n* = 4), *S.* Kentucky (*n* = 4), and *S.* Cerro (*n* = 4), see Additional file 1. *Salmonella* Typhimurium ATCC® 14028 was used as a positive control and its *sir*A isogenic mutant as a negative control. All isolates were susceptible to gentamicin as determined by antimicrobial susceptibility testing (MIC values between 0.25 and 1 μg/ml) by the Cornell University Animal Health Diagnostic Center. *Salmonella* isolates were grown on Luria Bertani (LB) plates at 37°C for 16 hours. A colony was transferred into 5 mL LB broth and incubated 18 hours at 37°C, without shaking. After 18 hours of incubation, 1 mL of each culture was pelleted by centrifugation and re-suspended in 1 mL of Phosphate Buffered Saline (PBS) pH 7.4. Bacterial cells were diluted and Caco-2 cells were inoculated at an MOI of 10. Each strain was inoculated in triplicate in each of the 3 experiments conducted. Appropriate dilutions were plated on LB for calculation of the initial inoculum.

For all the experiments Caco-2 cells were maintained in Dulbecco’s Modified Eagle Medium (DMEM) 20% FBS 1% non-essential amino acids at 37°C and 5.0% CO_2_, for no more than 50 passages. The 24-well plates were seeded at a concentration of 5.0 × 10^4^ cells/well and incubated at 37°C and 5% CO_2_ for 48 hours. Thirty minutes before the cells were inoculated with *Salmonella*, media in the 24-well plate was replaced with fresh media. Caco-2 cells were inoculated, and incubated at 37°C and 5% CO_2_ for 1 hour, followed by 3 washes with pre-warmed PBS. Fresh media was distributed into each well followed by a 15 minute incubation at 37°C and 5% CO_2_. Finally, media with gentamicin (50 μg/mL) was added and the cells were incubated for 1 hour at 37°C and 5% CO_2_. The cells were then lysed by vigorously pipetting 500 μL of chilled water in each well. The bacterial suspensions recovered were plated on LB and incubated at 37°C overnight. Invasion efficiency was calculated as [CFU recovered/CFU infected] × 100. Statistical analysis was performed using SAS software (SAS Institute Inc., Cary, NC, USA). The invasion efficiencies were analyzed using one-way analysis of variance (ANOVA), Tukey post hoc test, and the data was log-transformed to satisfy ANOVA assumptions of normality.

### Availability of supporting data section

All raw read files have been deposited in the Sequence Read Archive of the National Center for Biotechnology Information (http://www.ncbi.nlm.nih.gov/Traces/sra/) under entries PRJNA185435, PRJNA187190–187196, PRJNA187371, PRJNA187373, PRJNA187542, PRJNA187545, PRJNA187919–187921, PRJNA187962, PRJNA187963, PRJNA187965–187974, and PRJNA73959. The de novo assembly of strain FSL R8-0235 has been deposited as a Whole Genome Shotgun project at DDBJ/EMBL/GenBank under the accession JMIJ00000000. The version described in this paper is version JMIJ01000000.

## Electronic supplementary material

Additional file 1: **Isolates used in invasion assay.** Microsoft excel file listing isolates used in invasion assay. (XLSX 46 KB)
